# Point of Care Ultrasound-Diagnostic Approach of an Atypical Negative Pressure Pulmonary Oedema

**DOI:** 10.5152/TJAR.2022.21365

**Published:** 2022-08-01

**Authors:** André Silva, Inês Furtado, Beatriz Grenho, Marta Isidoro

**Affiliations:** 1 Department of Anaesthesia, Hospital Garcia de Orta, Almada, Portugal; 2 Department of Anaesthesia, Centro Hospitalar e Universitário do Algarve, Faro, Portugal

**Keywords:** Negative pressure pulmonary oedema, point of care ultrasound, pulmonary complications

## Abstract

A 42 years old patient who underwent laparoscopic appendectomy developed negative pressure pulmonary oedema (NPPO) with an atypical presentation. Point of care ultrasound (POCUS) played a decisive role in the diagnostic approach. POCUS, as a goal-orientated tool, can provide key elements for a prompt diagnosis of respiratory and airway complications in the surgical patient. The identification of a B-line pattern, as an imagiological surrogate of alveolar oedema, with a specific distribution and the exclusion of other differential diagnosis using POCUS allowed for an early NPPO diagnosis. NPPO is a rare, potentially life threatening complication whose early diagnosis and direct treatment may lead to better outcomes. Our case emphasizes the diagnostic role of ultrasound in the operating theatre in the identification of life threatening airway and pulmonary complications, such as NPPO.

**Main Points**

- Negative pressure pulmonary oedema may have an atypical presentation.
- Negative pressure pulmonary oedema diagnosis is supported by a lung ultrasound with the presence of a B-pattern on central and non-dependant areas.
- Point of care ultrasound (POCUS) can provide valuable data to guide the differential diagnosis of pulmonary perioperative complications.
- The role of POCUS in the operation room and post-anaesthesia care should be known and have to be enlightened among anaesthesiologists.

## Introduction

Negative pressure pulmonary oedema (NPPO) is a rare form of noncardiogenic pulmonary oedema that develops when negative intrathoracic pressure is generated against a closed upper airway. It has an incidence as low as 0.01%–0.1%,^1^ with the most common clinical presentation being airway obstruction on general anaesthesia emergence. The negative intrapleural pressure increases both pulmonary capillary permeability and hydrostatic pressure, favouring fluid translocation to interstitial and alveolar spaces, overcoming fluid drainage, and originating pulmonary oedema.^1,2^

Negative pressure pulmonary oedema diagnosis usually requires identification of an upper airway obstruction accompanied by acute respiratory distress symptoms, hypoxaemia, and traditionally a chest X-ray supporting it.^1^ Clinical severity varies and treatment includes oxygen and diuretic therapy, positive pressure ventilation, and extracorporeal membrane oxygenation.^3^

Point of care ultrasound (POCUS) is a bedside tool that can provide important information for the diagnoses of perioperative complications.^4^

When performing lung ultrasound, the presence of at least 3 B-lines within the same intercostal space is considered a positive B-pattern.^4^ This artefact is thought to be caused when a small amount of fluid is trapped between 4 aligned bubbles forming a tubular structure that is able to continuously oscillate when struck by ultrasound. This originates a strong vertical reverberation artefact that represents closely spaced horizontal echoes that are repeatedly emitted back to the transducer. These bubble-tetrahedron complexes can be formed when pathological processes alter de air-fluid dynamics of the interstitial space and alveoli.^5^ In pulmonary oedema, fluid tends to accumulate in the interlobular septa, until the point when it starts filling the alveoli that transition from air to fluid-filled state. This creates the subpleural conditions for the generation of B-lines.^6^ A diffuse B-pattern is sensitive for pulmonary oedema and its presence on central and non-dependent areas is consistent with NPPO as those areas have the lowest perivascular and interstitial pressure during inspiratory effort.^7^

We report a clinical case where a young healthy patient submitted to laparoscopic appendectomy developed NPPO with an atypical presentation. We aim to discuss the role of POCUS as a powerful tool to diagnose postoperative pulmonary complications such as NPPO.

We have obtained informed consent from the patient for the publication of this case report.

## Case Presentation

A 42-year-old man without any comorbidity, American Society of Anesthesiology I (ASA I), presented for an urgent laparoscopic appendectomy.

We performed a rapid sequence induction and anaesthesia maintenance with desflurane. Multimodal analgesia, antiemetics, and sugammadex were also administered at the end of an uneventful surgical procedure. Fully awake tracheal extubation was performed without complications.

After a few minutes, the patient suddenly exhibited desaturation to the lowest of 76% and depression of consciousness. Scarce pink frothy secretions and diffuse crackles on pulmonary auscultation were present. All the remaining vital signs and physical observation were normal. No airway obstruction was identified.

We applied face mask-assisted ventilation with 1.0 FiO_2_. The patient recovered, despite maintaining saturations of 90%-93% with 0.5 FiO_2_. Blood gas analysis revealed a PaO_2_ of 56 mm Hg with no other abnormalities.

**Figure 1.**
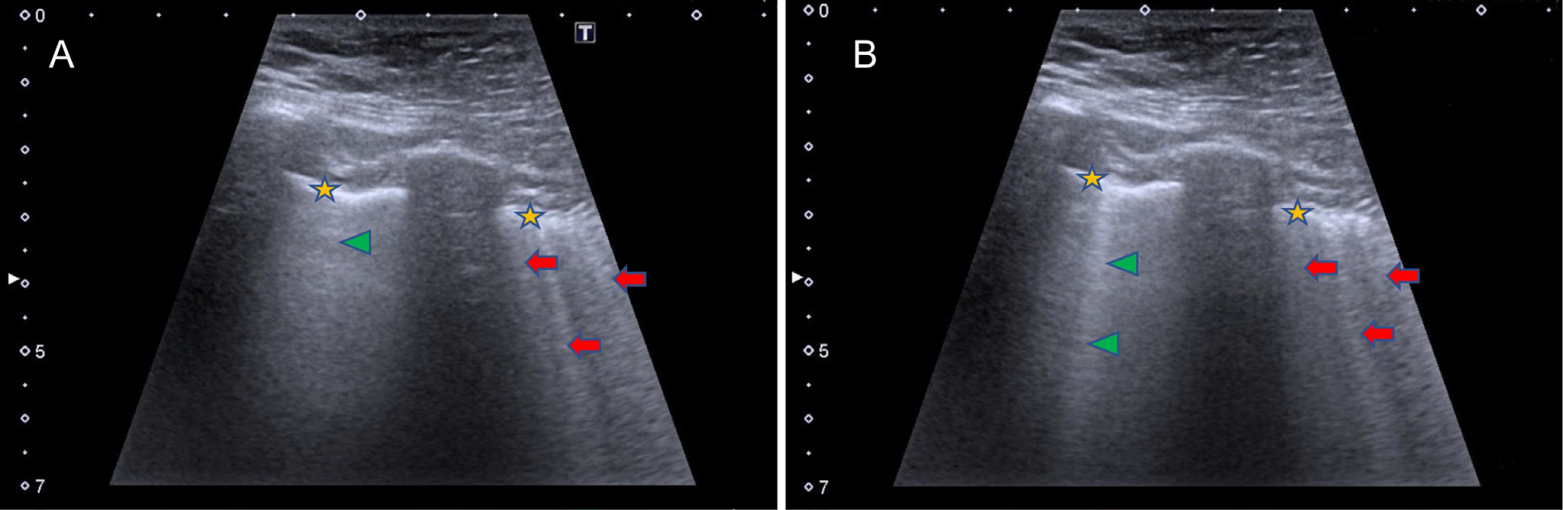
A-B. Lung ultrasound of right lateral upper lung field showing B-line pattern. Red arrows, B-Lines; Green arrowheads, coalescent B-Lines; Yellow stars, pleural line.

We performed POCUS assessment in the post-anaesthetic care unit. Lung ultrasound showed a diffuse B-line pattern (Figure 1), especially prominent in central and non-dependant areas. Lung sliding was present throughout lung fields. No hepatisation or pleural effusion was observed. Cardiac ultrasound showed normal biventricular size and function and absence of a D-sign. Inferior vena cava had normal diameter and over 50% collapsibility on inspiration.

NPPO diagnosis was assumed and the patient was placed under CPAP and furosemide.

**Figure 2.**
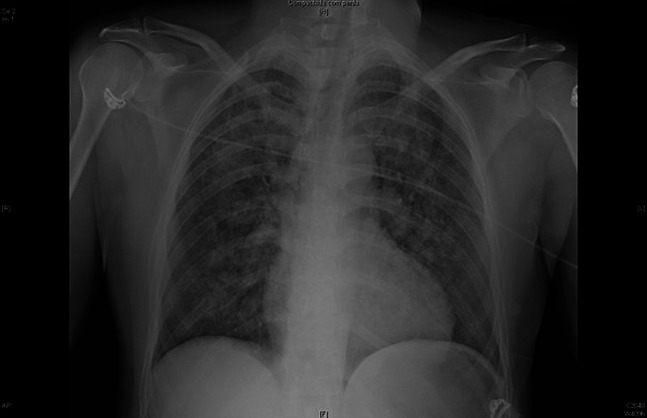
Antero-posterior chest radiography showing a diffuse interstitial and alveolar infiltrate.

Chest radiography confirmed a diffuse interstitial and alveolar infiltrate pattern (Figure 2).

A few hours later, oxygen saturation increased above 95% and the patient was transferred to the surgical ward where he recovered uneventfully until discharged home asymptomatic on the sixth postoperative day.

## Discussion

In our case, we believe that upon transfer to bed, the patient developed a sudden undiagnosed airway obstruction, probably due to upper airway collapse. A 42-year-old man without comorbidities may have generated large negative intrathoracic pressure with only a few breath cycles, enough to develop NPPO.

When NPPO diagnosis is not straightforward, POCUS may provide decisive information to support a prompt diagnosis and immediate treatment.

Point of care ultrasound is an easily accessible, simple, rapid, and goal-orientated tool that can play an important role in the perioperative management. Bedside cardiac, lung, abdominal, gastric, and airway ultrasound have been described.^8^ Lung ultrasound has been shown to be a valuable tool in the determination of causes of hypoxia. Normal ultrasound usually presents with lung sliding and A-line patterns and less than 3 B-lines per intercostal space.^4^

The presence of 3 or more B-lines in the same intercostal space suggests a fluid-filled alveolo-interstitial space. In our report, the B-Line pattern was especially noticeable in central and non-dependent areas, consistent with NPPO.^4^

Ultrasound also enabled the exclusion of pneumothorax since we observed lung sliding throughout the fields, B-lines, and lung pulse in combination with the absence of lung point.^8^

Other diagnosis such as pleural effusion and atelectasis were also excluded by the absence of hypoechoic fluid around the lung base and lung hepatisation, respectively.

A rapid cardiac exam can also provide information about cardiogenic pulmonary oedema. Biventricular size and wall motion, pleural effusions and inferior vena cava size, and collapsibility can be useful surrogates of cardiac function.^4^

Although the role of POCUS in areas of critical care and emergency medicine is well consolidated, in anaesthesiology, the use of ultrasound on a regular basis is more associated with peripheral nerve blocks and placement of vascular catheters.^4^ Whole-body POCUS incorporation into modern anaesthesiology is more recent and its full potential as an adjunct to traditional diagnostic tools is still to be known.

To the best of our knowledge, this is the first report of POCUS use in the differential diagnosis of NPPO.

## Conclusion

Point of care ultrasound´s role as a perioperative management tool is still evolving. Our case emphasises how this easily available bedside exam enables the early identification of perioperative pulmonary complications, such as NPPO. We believe that the full potential of POCUS in the management of perioperative complications should be enlightened and known among anaesthesiologists.
